# Human Papillomavirus (HPV) Infection in Early Pregnancy: Prevalence and Implications

**DOI:** 10.1155/2019/4376902

**Published:** 2019-03-24

**Authors:** Deeksha Pandey, Vani Solleti, Gazal Jain, Anwesha Das, Kabekkodu Shama Prasada, Shobha Acharya, Kapaettu Satyamoorthy

**Affiliations:** ^1^Department of Obstetrics and Gynecology, Kasturba Medical College, Manipal Academy of Higher Education, Manipal, India; ^2^School of Life Sciences, Manipal Academy of Higher Education, Manipal, India

## Abstract

**Introduction:**

Young women (20-35 years) are at high risk of HPV infection, although the majority of the infections are asymptomatic and are cleared spontaneously by the host immune system. These are also the group of women who are sexually active and are in the population of pregnant women. During pregnancy, the changes in the hormonal milieu and immune response may favor persistence of HPV infection and may aid in transgenerational transmission thereby furthering the cancer risk. In the present study, we determined the prevalence of vaginal HPV infection in early pregnancy and attempted to relate with pregnancy outcome.

**Material and Methods:**

Vaginal cytology samples were collected from the condoms used to cover the vaginal sonography probe during a routine first trimester visit to the hospital. All women were followed up throughout pregnancy and childbirth. Maternal and neonatal outcomes were recorded.

**Results:**

We found a prevalence of HPV infection around 39.4% in our population. Interestingly all HPV positive women were infected with one or more high risk HPV viruses with an overlap of intermediate and low risk in 43% and 7.3%, respectively. Women with preterm prelabor rupture of membranes (PPROM) showed a statistically higher incidence in HPV positive (7.3%) group as compared to the HPV negative (3.2%) group.

**Conclusion:**

The prevalence of genital HPV infection is high during pregnancy (around 40%) and was associated with higher incidence of PPROM.

## 1. Introduction

Human Papillomavirus (HPV) is the most common sexually transmitted viral infection. More than 100 human types of HPV are identified which are known to infect epithelial cells including skin, respiratory mucosa, or the genital tract. HPV types specific to genital tract infections are classified into three risk categories; low risk (6, 11, 40, 42, 43, 44), intermediate (31, 33, 35, 51, 52), and high-risk (16, 18, 45, 56) types based on their relative malignant potential [1, 2].

Young women (20-35 years) are at maximum risk of HPV infection, although majority of these are asymptomatic and get cleared spontaneously because of strong immune system. This is the age when women are more sexually active. In the developing nations this age group forms a major cohort among the pregnant population. During pregnancy the changed hormonal milieu and immune response might favor presence or persistence of HPV infection.

A systematic review of literature demonstrated a wide variation in the prevalence of HPV in pregnant women from 5.5 to 65% [3]. The high prevalence in pregnancy can be attributed to changed hormonal milieu and decreased immunity. In several populations, various HPVs have been found to be associated with preterm rupture of membranes (PROM), preeclampsia, fetal growth restriction (FGR), preterm delivery, and placental abnormalities [4–6]. No such data is available from the Indian subcontinent reporting association of HPV with pregnancy and its outcome.

In the present study, we determined prevalence of vaginal HPV infection in early pregnancy and its correlation with the pregnancy outcome.

## 2. Material and Methods

This prospective study was conducted in a University Teaching Hospital over a twenty-month period (November 2015–June 2016). The study protocol was approved by institutional review board (IEC127/2016). The study cohort included random obstetric population who presented to us in first trimester (up to 14 weeks) with singleton pregnancy and were planning to continue in one centre throughout pregnancy and delivery. Written informed consent was obtained from all participants. Those women who were previously diagnosed with HPV infection or were detected to have abnormal cervical cytology in Pap smear tests were excluded from the study.

### 2.1. Sample Collection

Vaginal cytology samples were collected from the condoms used to cover the transvaginal sonography (TVS) probe during a routine first trimester scan. Condoms used to cover the TVS probe were used, as regular speculum examination in asymptomatic pregnant women is not a part of routine antenatal care in our set-up and it is not acceptable to many pregnant women. However every patient undergoes a TVS in first trimester either for confirmation or dating of first trimester early anomaly screening. Condom thus obtained after TVS was immediately put in a 50 cc sterile plastic container with phosphate buffer saline solution (PBS), vigorously shaken, and condom was discarded. The solution was kept at 4°C and processed within one hour. All women were followed up throughout pregnancy and childbirth and maternal and neonatal outcomes were recorded.

### 2.2. DNA Extraction

Tubes were centrifuged at 2000rpm for 10min and the pelleted cells were digested with DNA extraction buffer and proteinase k followed by standard phenol chloroform and ethanol precipitation method. The quality and quantity of the DNA were assessed by agarose gel electrophoresis and spectrophotometrically and stored at -20°C until used. On an average we could extract 200-250 ng of DNA from the condom which was used to cover the TVS probe during first trimester ultrasonography.

### 2.3. HPV Typing

HPV DNA testing was performed as published earlier. In brief, 100-200 ng of DNA was amplified by nested PCR using PGMY09/11 and GP5+/GP6+ primers with beta globin as internal control. PCR product was gel eluted, purified, and sequenced using Big Dye terminator kit (ABI, USA) in Genetic Analyzer 3130XL (ABI, USA). The HPV types were identified by NCBI BLAST search. The PCR was performed using appropriate positive and negative controls. The samples were scored as negative after two rounds of independent testing.

HPV positivity and type were then correlated with various maternal and fetal variables and pregnancy outcomes.

### 2.4. Statistical Method

The data on categorical variables is shown as n (% of cases) and the data on continuous variables is presented as Mean and Standard deviation (SD) across two study groups. The intergroup comparison of categorical variables is performed using Chi-square test/Fisher's exact probability test. The statistical significance of intergroup difference of mean of continuous variables is tested using independent sample ‘t' test or unpaired ‘t' test. The underlying normality assumption was tested before subjecting the study variables to ‘t' test. The entire data is entered and cleaned in MS Excel before its statistical analysis.

The p-values less than 0.05 are considered to be statistically significant. All hypotheses were formulated using two tailed alternatives against each null hypothesis (hypothesis of no difference). The entire data is statistically analyzed using Statistical Package for Social Sciences (SPSS ver 21.0, IBM Corporation; NY, USA) for MS Windows.

## 3. Results

**Figure 1:**
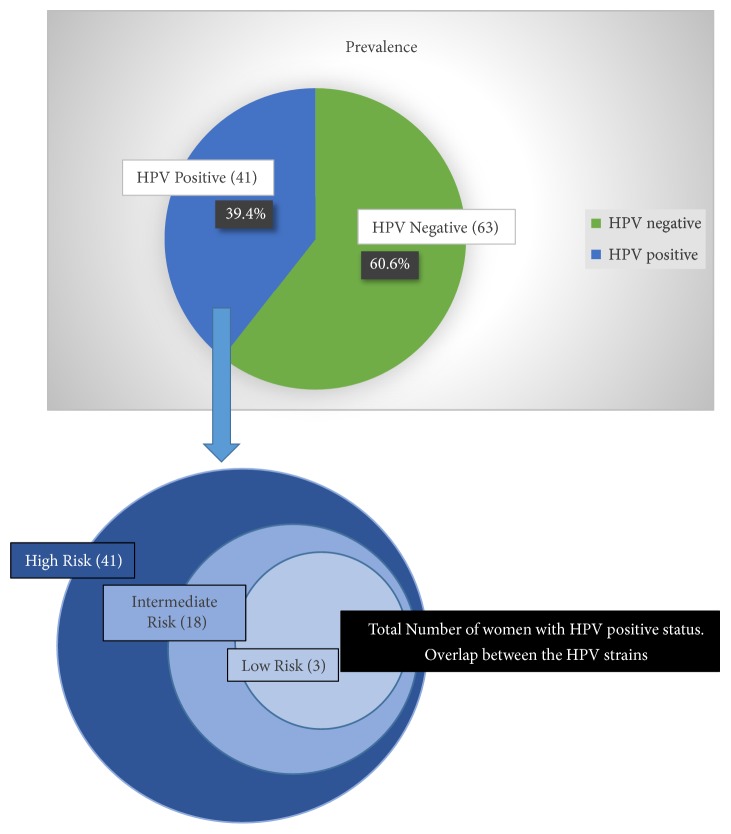
Prevalence of HPV infection in the study population and distribution of high, intermediate, and low risk HPV virus in the samples.

A total of 115 samples were collected; however 104 samples completed the study. HPV prevalence: in our study cohort out 41/104 (39.4 %) were HPV positive, while 63/104 (60.6%) were found to be negative from HPV vaginal infection. Interestingly, typing of the HPV positive samples indicated that all these 41 samples were positive for high-risk HPV types (HPV 16, HPV18, HPV45, HPV56, and HPV97). Eighteen of them showed evidence for coinfection with intermediate risk HPV types such as HPV 87, HPV35, HPV51, HPV52, and HPV82, while only 3 samples were coinfected with low risk HPV (HPV29, HPV32, HPV54, HPV61, HPV84, and HPV87). Most frequent HPV types in our cohort were HPV45 (60%), followed by HPV18 (48.8%) and HPV16 (43.9%) (Figure 1).

HPV status and pregnancy outcome: in order to compare the pregnancy outcome in HPV positive versus HPV negative women, we analyzed the basic demography in the two groups. The mean age (HPV positive: 28.1±4.6 years; HPV negative: 27.3± 3.6 years) and mean number of pregnancies (HPV positive: 1.7± 1; HPV negative: 1.7± 0.9) were comparable in both of the groups, while the years of married life in HPV positive pregnant women were 3.6±2.7 years and they were 2.8±2.7 years in HPV negative women (p<0.05) (Table 1).

On comparing the pregnancy complications (Table 2) between the two groups, we could not find a statistically significant difference between abortions, gestational diabetes, and hypertension or fetal growth restriction. Preterm prelabor rupture of membranes (PPROM) however showed a statistically higher incidence in HPV positive (7.3%) group, as compared to the HPV negative (3.2%) group. HPV status however did not differ between the two groups in terms of gestational age at delivery, birth weight, and Apgar scores at 1 minute and 5 minutes (Table 3).

## 4. Discussion

We found HPV prevalence of around 39.4% in pregnant women in our population. Interestingly all HPV positive women were infected with one or more high risk HPV viruses with an overlap of intermediate and low risk in 43 % and 7.3%, respectively. HPV positive status was seen more frequently in women with longer married life (which might have an indirect correlation with years of sexual activity) and higher BMI.

HPV infection is common among sexually active young adults, with an estimated prevalence between 20.0% and 46.0% [7]. During pregnancy the risk is expected to be high owing to the fact that pregnancy is a kind of suppressed immunity state [8]. A prospective study in Brazil also found a higher prevalence of HPV infection among pregnant women (25.3%) as compared to the nonpregnant women (13%) [9].

In African pregnant women prevalence of HPV DNA was 33.3%. Around 62% of HPV-positive women were infected with high risk and/or possible or probable high risk (pHR) also known as intermediate risk HPV types. The five most prevalent HPV types in this study were HPV-52 and HPV-67, HPV-53, HPV-45, and HPV-18. HPV-16 was rare (1.2%) [10].

HPV in pregnancy is interesting and important to study for two reasons. First vaginal infections are known to adversely affect pregnancy outcome. Secondly HPV infection of the placenta is possible as trophoblast cells appear to have the machinery for HPV replication [11, 12] and placental affliction can directly cause foetal growth retardation, preeclampsia, abortions, and preterm births.

In our cohort only the incidence of PPROM was found to be more among HPV positive pregnant women as compared to those who were HPV negative. There was no difference in risk of abortions or preterm births (without PPROM). In other studies too there is equivocal evidence for placental infection with HPV causing placental dysfunction leading to spontaneous abortions and preterm births [11, 13]. A recent study concluded that maternal HPV infection is not a risk factor for preterm birth or pregnancy related hypertension [14]. However, in this study the presence of HPV during pregnancy was not studied. A retrospective analysis was performed for women after delivery who had cervical cancer screening test results within three years before the childbirth. In light of these, the results must be interpreted keeping in mind that new infections can be detected in pregnancy because of changed hormonal and immune milieu and the transient nature of HPV infection is also common in young women [8, 15]. Another retrospective study found a 2-fold increased risk of preeclampsia in women who had high risk HPV status at an entry to prenatal care compared with those with at least 2 normal pap smears [6].

As per our knowledge, this is the first prospective study from Indian subcontinent to find out the prevalence of HPV in our population during pregnancy and its effect on pregnancy and childbirth. The factors affecting HPV infection in our sociocultural environment might be different. Furthermore, though cervical cancer screening with cytology is available all over India, it has not yet become practical for population screening in our country. Thus this kind of simple effortless collection of samples for screening, in the target population during pregnancy, is an interesting area to study further. However, the cost of HPV testing needs to be justified if it is done beyond the periphery of research. HPV screening during pregnancy might also help to optimise our goals towards a better reproductive and child health care, as we found it to be associated with poor pregnancy outcomes. Another novelty of this study was the technique with which we collected samples from the condoms which were used to cover the TVS probe. No other study has mentioned this technique of sample collection and its feasibility until date, in scientific English literature.

Small size of study population is the limiting factor here. Other limitations include only one time testing in first trimester for HPV status. New HPV infection towards term or clearance of HPV before delivery would be interesting to study and its correlate with pregnancy outcomes might be more meaningful, as would be the study of the vertical transmission rate by studying the placental affliction and HPV status of the newborn.

## 5. Conclusion

The prevalence of genital HPV infection is high (40%) during pregnancy and was associated with higher incidence of PPROM in our study. Condom used to cover TVS probe during sonographic evaluation of pregnancy can be used to collect samples for HPV detection where sociocultural belief prohibits direct speculum examination during routine antenatal care in asymptomatic women.

## Figures and Tables

**Table 1:** Comparison of basic demography between the HPV positive and negative groups.

Demographic characteristics	HPV Positive	HPV Negative	P value
	(n= 41)	(n= 63)	
Age in years (mean ± SD)	28.1±4.6	27.3± 3.6	0.708
Marital Life in years (mean ± SD)	3.6±2.7	2.8±2.7	0.046
Number of Pregnancies (mean ± SD)	1.7± 1	1.7±0.9	0.215
BMI (mean ± SD)	22.4±3.7	21±2.8	0.023

**Table 2:** Pregnancy complications in HPV positive versus negative group.

Pregnancy Complications	HPV Positive	HPV Negative	P value
	(n= 41)	(n= 63)	
Abortions (%)	2 (4.8%)	3(4.7%)	0.100

Pregnancy Related Hypertension (%)	3 (7.3%)	4(6.3%)	0.470

Gestational Diabetes (GDM) (%)	2 (4.8%)	4(6.3%)	0.641

Foetal Growth Restriction (FGR) (%)	2 (4.8%)	3(4.7%)	0.100

Preterm Pre-labor Rupture of Membranes (PPROM) (%)	6 (14.6%)	2 (3.2%)	0.026

Preterm Delivery (%) Spontaneous – Excluding PPROM	3 (7.3%)	2 (3.2%)	0.324

**Table 3:** Neonatal outcome in HPV positive versus negative group.

Neonatal Outcome	HPV Positive	HPV Negative	P value
	(n= 41)	(n= 63)	
Gestational age at Delivery (in Weeks) (mean ± SD)	38.4±0.4	39.2±05	0.162

Birth Weight (in grams) (mean ± SD)	3010±750	2970±630	0.461

Apgar at 1 min (Median)	9	9	--

Apgar at 5 min (Median)	9	9	--

## Data Availability

The data used to support the findings of this study are available from the corresponding author upon request.
